# Claude Syndrome: A Rare but Easily Recognizable Midbrain Lacunar Syndrome

**DOI:** 10.7759/cureus.87505

**Published:** 2025-07-08

**Authors:** Octavio Carranza, Roxana M Dragomir, Jessica Canosa, Marc A Swerdloff

**Affiliations:** 1 Neurology, Florida Atlantic University Charles E. Schmidt College of Medicine, Boca Raton, USA; 2 Marcus Neuroscience Institute, Boca Raton Regional Hospital, Boca Raton, USA

**Keywords:** claude syndrome, isolated midbrain infarction, midbrain stroke, posterior circulation stroke, stroke

## Abstract

Claude syndrome (CS) is a rare and classic neurological condition resulting from an isolated midbrain infarction (IMI). Its clinical presentation and anatomical localization remain subjects of ongoing debate in the literature. We present the case of a patient evaluated at our stroke center, with neuroimaging and video documentation of her resolving deficits. In addition, we provide a review of the syndrome’s history, epidemiology, clinical features, and potential etiologies.

## Introduction

Claude syndrome (CS) was first described in 1912 by the French psychiatrist and neurologist Henri Claude (1869-1945) [1]. His original case involved a house painter who developed left arm and leg asynergy and ataxia, along with severe gait instability [1]. The patient exhibited left lateral pulsion, left-hand dysdiadochokinesia, complete ptosis of the right eyelid, and ophthalmoplegia due to a right oculomotor nerve palsy. Muscle strength and sensory function were preserved. Postmortem examination revealed a paramedian mesencephalic infarction involving the crossing fibers of the superior cerebellar peduncles, the medial half of the red nucleus, and the fascicles of the oculomotor nerve. The medial longitudinal fasciculus and substantia nigra were also involved, while the cerebral peduncles and medial lemniscus were spared [2]. In Claude's original description, there was no mention of fourth cranial nerve palsy. However, about a decade later, he expanded the syndrome to include fourth cranial nerve palsy and hemisensory loss. This change contributed to confusion in the literature. Variants such as a pupil-sparing form, ipsilateral hemiataxia [3], and even bilateral CS [4] have since been reported. Despite these variations, CS is now generally recognized as a midbrain infarction (MI) presenting with oculomotor nerve palsy and contralateral appendicular or truncal ataxia, attributed to cerebellar rather than rubral tremor.

The precise anatomical localization of the lesion in CS has been a topic of debate for decades. In Henri Claude's original description, the lesion involved the medial half of the red nucleus and the decussation of the superior cerebellar peduncle. Most neuro-ophthalmology textbooks attribute the tremor associated with CS to involvement of the red nucleus. However, in a case series analyzing radiological and pathological findings from six patients with CS, along with a review of 18 previously reported cases, the authors found a different pattern [5]. Pooled MRI and pathological data identified lesions predominantly in the midbrain tegmentum, just beneath the red nucleus, implicating the superior cerebellar peduncle fibers. Notably, only 3 of the 18 cases had additional lesions involving the red nucleus. These findings support the view that the superior cerebellar peduncle is the primary structure responsible for the tremor and ataxia characteristic of CS.

Midbrain infarcts can present with a wide range of symptoms. Most commonly, they involve a complete or partial palsy of the third cranial nerve, accompanied by sensory, motor, or cerebellar deficits. In addition to CS, several other eponymous syndromes are associated with MIs. In 1889, Moritz Benedikt (1835-1920) described a syndrome characterized by oculomotor palsy, contralateral hemiparesis, tremor, and involuntary movements due to involvement of the oculomotor nerve, red nucleus, substantia nigra, and the cerebral peduncle [6,7]. Earlier, in 1863, Herman Weber (1823-1918) reported oculomotor nerve palsy with contralateral hemiparesis secondary to a lesion in the midbrain cerebral peduncle [8]. In 1879, Hermann Nothnagel (1841-1905) described bilateral oculomotor palsy of varying degrees and gait ataxia caused by lesions involving the quadrigeminal plate, affecting both the superior and inferior colliculi [9]. The German anatomist Friedrich C.G. Wernekinck (1798-1839) was the first person to describe the presence of a decussation of the superior cerebellar peduncles. Damage to the area of decussation before their entrance into the red nucleus (Wernekinck commissure) results in cerebellar dysarthria, ataxic gait, truncal and bilateral appendicular ataxia, from bilateral cerebellar dysfunction [10,11]. The medial longitudinal fasciculus can be involved, causing internuclear ophthalmoplegia. On occasion, damage to the dentatorubral fibers, a component of the triangle of Guillain-Mollaret, causes palatal myoclonus. Although the syndrome carries his eponym, this was first described by Lhermitte in 1958 [12]. Parinaud syndrome results from compression of the superior tectal plate, resulting in upward gaze palsy, convergence-retraction nystagmus, and pupillary light-near dissociation [13]. Additional features may include bilateral upper eyelid retraction and downbeat nystagmus. Parinaud syndrome was named after the French ophthalmologist Henri Parinaud (1844-1905), who described it in 1883 [14].

Although midbrain syndromes have been well-recognized for decades, the literature regarding their clinical features and neuroanatomical correlations remains complex and, at times, confusing. In this report, we present the case of a patient diagnosed with CS at our institution. We describe her symptoms, physical examination findings, and imaging results, followed by a brief review of the relevant literature.

## Case presentation

**Figure 1:**
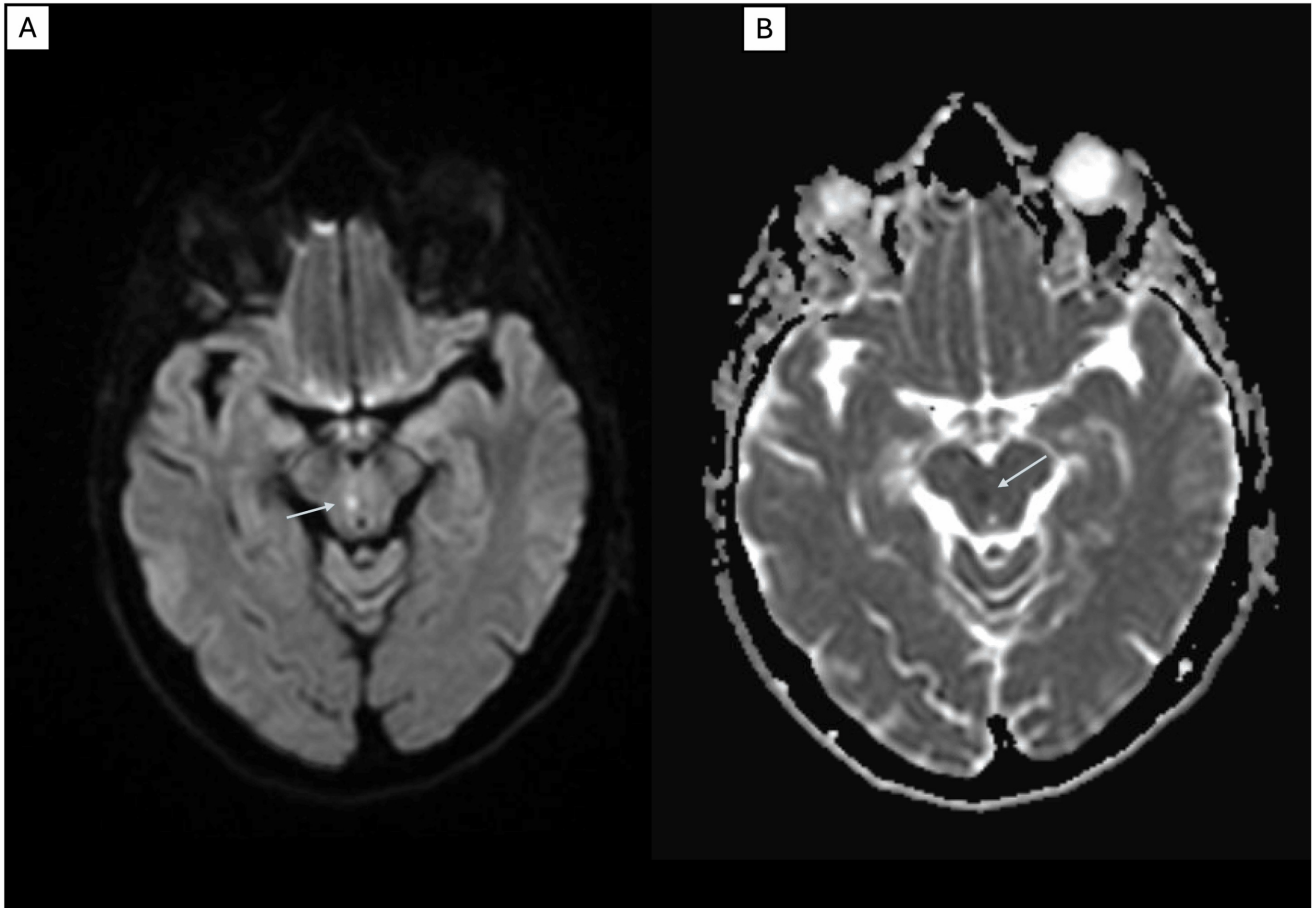
Right paramedian midbrain stroke demonstrated by MRI of the brain Axial images at the level of the midbrain. (A) Diffusion-weighted imaging (DWI) sequence. (B) Apparent diffusion coefficient (ADC) sequence. Restricted diffusion is evident in the right paramedian midbrain region on both DWI and ADC sequences, consistent with an acute lacunar midbrain stroke (arrows).

**Figure 2:**
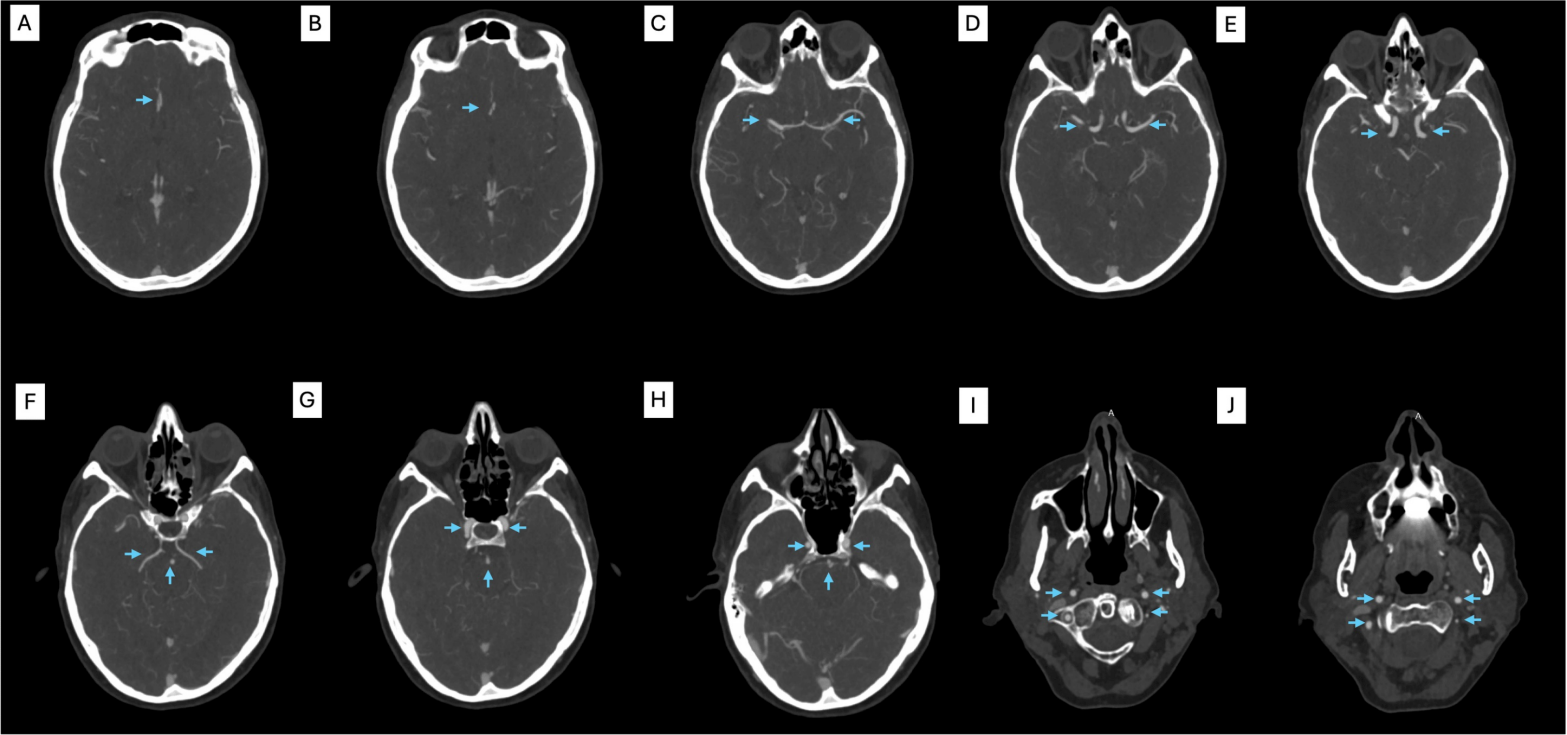
CT angiography showing no flow-limiting stenosis in the anterior or posterior circulation (A, B) Anterior cerebral arteries. (C, E) Middle cerebral arteries and internal carotid arteries. (F) Posterior cerebral arteries and basilar artery. (G, H) Carotid arteries and basilar arteries. (I, J) Carotid arteries and vertebral arteries.

A 68-year-old woman presented to the emergency department with a one-week history of intermittent vertigo accompanied by vertical diplopia. She noted that her symptoms worsened when looking to the left. The day before admission, she experienced persistent vertical binocular diplopia and an unsteady gait. Her medical history included hyperlipidemia and recurrent giant cell arteritis (in remission since episodes in 2017 and 2023). Initial physical examination revealed nystagmus on right horizontal gaze and right eyelid ptosis. There were no focal motor or sensory deficits. The head impulse was negative for corrective saccade, and there was no vertical ocular skew deviation or hearing loss. Finger-to-nose and heel-to-shin testing did not demonstrate dysmetria. She was able to ambulate without signs of ataxia at the time of initial evaluation. A non-contrast computed tomography (CT) scan of the head showed no acute infarct, hemorrhage, or mass. Magnetic resonance imaging (MRI) of the brain revealed a punctate area of restricted diffusion just to the right of the midline in the midbrain (Figure 1). CT angiography of the head and neck did not demonstrate any vessel occlusion or stenosis (Figure 2). The patient was discharged on a 21-day course of dual antiplatelet therapy, followed by aspirin 81 mg daily and a high-intensity statin. At follow-up one week later, examination revealed a right partial third nerve palsy with double elevator paresis, right medial rectus paresis causing exodeviation, and left upper extremity dysmetria (Video 1).

**Video 1:** Clinical examination of the patient with Claude syndrome at the first post-discharge visit Note: Written informed consent was obtained from the patient.

## Discussion

The brainstem is a compact structure located at the base of the brain, responsible for numerous vital functions. It is divided into three parts (the midbrain, pons, and medulla) and is centrally positioned within the posterior fossa of the cranial cavity. The brainstem contains all major sensory, motor, and cerebellar tracts, as well as the majority of cranial nerve nuclei and pathways. Additionally, it houses critical nuclei involved in regulating consciousness and arousal, muscle tone and posture, and cardiovascular and respiratory homeostasis.

Anatomically, the brainstem is bounded rostrally by the midbrain-diencephalic junction (where the midbrain connects to the thalamus and hypothalamus) and caudally by the cervicomedullary junction at the level of the foramen magnum. The decussation of the corticospinal tract occurs in the caudal medulla. The brainstem may be functionally and anatomically subdivided at the pontomesencephalic junction (midbrain-pons) and the pontomedullary junction (pons-medulla oblongata) [15]. 

The midbrain is further subdivided into the dorsal tectum, which contains the paired superior and inferior colliculi (collectively known as the corpora quadrigemina), the midbrain tegmentum, and the cerebral peduncles. The superior colliculi process visual information, whereas the inferior colliculi are involved in auditory processing. Ventral to these structures lie multiple nuclei surrounding the cerebral aqueduct, including the pretectal, oculomotor, Edinger-Westphal, trochlear, reticular, and red nuclei. The midbrain tegmentum houses major ascending and descending fiber tracts, and the cerebral peduncles, bounded superiorly by the substantia nigra and contain the corticopontine and corticospinal tracts. The midbrain receives blood supply from branches of several arteries, including the basilar, posterior cerebral, superior cerebellar, posterior communicating, and both anterior and posterior choroidal arteries [16].

Isolated midbrain infarctions (IMIs) are rare. In the largest reported case series, Sheetal et al. [17] identified 520 cases of IMI, accounting for 9.2% of all posterior circulation strokes. This finding is consistent with another case series of 81 posterior circulation strokes, in which 8% were IMIs [15]. In the series by Sheetal et al., CS was the most common (43.8%) IMI presentation, followed by Wernekinck commissure syndrome (18.3%), Weber syndrome (8.3%), Nothnagel syndrome (8.3%), and Parinaud syndrome (6.3%). Benedikt syndrome was the least common, with no cases reported in two major series [18].

The midbrain is supplied by multiple arterial territories (as mentioned previously), which increases the likelihood of its involvement in infarctions affecting adjacent structures. Dorsal midbrain lacunes are typically caused by occlusion of penetrating branches from the basilar artery. The anterior midbrain is often affected in cases of atherosclerosis involving the P1 segment of the posterior cerebral artery. Embolism and arterial dissection are less common causes of midbrain stroke [19,20]. In the case series by Sheetal et al., small vessel disease accounted for the majority (47.9%) of IMIs [17]. Large vessel disease was identified in 27.1% of cases and cardioembolism in 10.4%. Common risk factors for lacunar strokes included hypertension (81.3%), diabetes (62.5%), hyperlipidemia (52.1%), and coronary artery disease (22.9%).

Claude syndrome may also occur in non-stroke contexts, such as neurocysticercosis [21] or posterior cerebral artery stenosis [22]. MRI enables classification of midbrain infarctions into four types based on anatomical location: anteromedial, anterolateral, lateral, and posterior. Anteromedial midbrain strokes frequently involve oculomotor disturbances; anterolateral midbrain strokes cause contralateral hemiparesis; lateral midbrain strokes manifest with contralateral pure hemisensory loss; while posterior midbrain strokes present with vertical gaze palsy and convergence retraction nystagmus. A minority of patients exhibit infarctions involving both posterior and paramedian territories.

Table 1 summarizes the eponymous midbrain stroke syndromes, their clinical findings, MRI localization, and the neuroanatomical structures involved.

**Table 1:** Midbrain stroke syndromes Summary of eponymous midbrain stroke syndromes with corresponding clinical features and lesion localization based on brain MRI findings.

Eponym	Structures involved	Clinical findings	MRI anatomical classification
Claude syndrome	Oculomotor nucleus, superior cerebellar peduncle, ± red nucleus	Ipsilateral oculomotor nerve palsy, contralateral cerebellar ataxia	Anteromedial midbrain
Benedikt syndrome	Oculomotor nucleus, red nucleus/superior cerebellar peduncle, corticospinal tract	Ipsilateral oculomotor nerve palsy, contralateral hemiparesis, contralateral involuntary movements/ataxia	Anteromedial midbrain
Weber syndrome	Oculomotor nerve, corticospinal tract	Ipsilateral oculomotor nerve palsy, contralateral hemiparesis	Anterolateral midbrain/lateral midbrain
Nothnagel syndrome	Oculomotor nerve nucleus, quadrigeminal plate (superior and inferior colliculi)	Bilateral oculomotor nerve palsy, truncal cerebellar ataxia	Posterior midbrain
Wernekinck syndrome	Decussation of both superior cerebellar peduncles (Wernekinck commissure)	Bilateral appendicular cerebellar ataxia, truncal cerebellar ataxia	Posterior midbrain
Parinaud syndrome	Vertical gaze center, posterior commissure, pretectal nuclei, oculomotor nerve fascicles, cerebral aqueduct	Upward gaze palsy, convergence-retraction nystagmus, pupillary light-near dissociation, bilateral upper eyelid retraction, downbeat nystagmus, obstructive hydrocephalus	Posterior midbrain

Midbrain strokes can result in varying degrees of disability after the acute phase, which is commonly assessed using the Modified Rankin Scale [23]. The scale ranges from 0 to 6, where 0 indicates no symptoms; 1, no significant disability despite symptoms, with the ability to perform usual activities; 2, slight disability, unable to carry out all previous activities but able to manage personal affairs without assistance; 3, moderate disability, requiring some help but able to walk unassisted; 4, moderately severe disability, unable to walk or attend to bodily needs without assistance; 5, severe disability, bedridden, incontinent, and requiring constant nursing care; and 6, death. Based on existing case series, 81.2% patients with IMIs have a Modified Rankin Scale of 0-1 at the end of three months, indicating no or minimal disability at follow-up.

## Conclusions

Midbrain strokes are an uncommon type of stroke that can present with a variety of clinical syndromes. CS is the most common form of midbrain stroke and is characterized by crossed findings of an oculomotor palsy and contralateral limb dysmetria. It is most commonly caused by a lacunar or small vessel infarction secondary to uncontrolled cardiovascular risk factors such as diabetes, hypertension, and hyperlipidemia. In our case, the patient presented with a lacunar midbrain stroke, likely due to small vessel disease. 

Historically, midbrain strokes have been described based on their clinical presentation. Numerous eponyms have been used in the neurological literature to describe the wide range of signs and symptoms associated with these strokes. However, there remains significant confusion regarding the relationship between these eponymous names and the actual clinical findings. In this article, we provided a comprehensive review of the original clinical description of these syndromes. MRI imaging allows for the anatomical classification of isolated midbrain infarctions into anteromedial, anterolateral, lateral, and posterior types. We believe this anatomical approach reduces the ambiguity associated with clinical terminology. Based on this classification, CS would most commonly correspond to an anteromedial isolated midbrain infarction.
